# Characterizing the roles of race and ethnicity in late‐onset Alzheimer's disease

**DOI:** 10.1002/alz70855_104897

**Published:** 2025-12-23

**Authors:** Razaq O Durodoye, Timothy H Ciesielski, Penelope Benchek, Jacquelaine Bartlett, Xiaofeng Zhu, Farid Rajabli, Giuseppe Tosto, Jonathan L Haines, William S Bush, Scott M Williams

**Affiliations:** ^1^ Case Western Reserve University School of Medicine, Cleveland, OH, USA; ^2^ Case Western Reserve Univeristy, Cleveland, OH, USA; ^3^ Case Western Reserve University, Cleveland, OH, USA; ^4^ John P. Hussman Institute for Human Genomics, University of Miami Miller School of Medicine, Miami, FL, USA; ^5^ Department of Neurology, College of Physicians and Surgeons, Columbia University, and the New York Presbyterian Hospital, New York, NY, USA

## Abstract

**Background:**

Race and ethnicity (R/E) are common descriptors used to stratify participants in genetic studies of late‐onset Alzheimer's disease (LOAD). These categories allow researchers to identify risk alleles, document effect size variation across groups, and asses etiologic differences among R/E groups. Importantly, R/E can serve as a proxy for unmeasured extrinsic risk factors correlated with, but separate from genetics, including the experience of R/E and the non‐medical drivers of health (NMDH). Using data provided by the Alzheimer's Disease Genetic Consortium (ADGC), we estimated the non‐genetic contributions of R/E on LOAD risk by adjusting for genetic risk factors and ancestry.

**Method:**

We obtained and cleaned genetic and demographic data from 42,015 ADGC participants (3,196 East Asian (EAS), 31,105 non‐Hispanic White (NHW), 1,646 Hispanic and Latino (HIS), and 6,068 non‐Hispanic Black (NHB) participants). To determine the risk contributions of NMDH captured by R/E, we performed logistic regression adjusting for *APOE* genotype, age, sex, and genetic ancestry via principal components. We next used K‐means clustering to identify genetically distinct groups and reran the previously defined regression models using these clusters in place of R/E designations.

**Result:**

Using NHB as the referent group, we found that EAS, NHW, and HIS all had significantly increased chances of LOAD (odds ratio (OR): 2.43, 2.07, 2.04, respectively; all *p* <0.05). Subsequent analyses rotating the R/E referent group revealed significant OR differences between most of the remaining R/E groups. Interestingly, the HIS OR did not differ from the EAS or NHW OR. K‐means clustering indicated the presence of five distinct genetic ancestries (African, East Asian, European, and two groups making up the HIS R/E group from the initial analyses ‐ Amerindian and genetically admixed Latino). Amerindian ORs significantly differed only from African ORs while the OR difference between genetically admixed Latino and East Asian was non‐significant. The remaining OR comparisons were identical to their respective R/E groups. Despite adjustment for genetic risk factors, R/E remained significant, indicating that R/E captures risk beyond that of genetic contributions.

**Conclusion:**

This report demonstrated that extrinsic factors, including NMDH captured through R/E independently associate with LOAD risk.

